# Antiangiogenic and antihepatocellular carcinoma activities of the *Juniperus chinensis* extract

**DOI:** 10.1186/s12906-016-1250-6

**Published:** 2016-08-08

**Authors:** Zong-Keng Kuo, Mei-Wei Lin, I-Huang Lu, Hsin-Jan Yao, Hsin-Chieh Wu, Chun-Chung Wang, Shyh-Horng Lin, Si-Yuan Wu, Tien-Soung Tong, Yi-Cheng Cheng, Jui-Hung Yen, Ching-Huai Ko, Shu-Jiau Chiou, I-Horng Pan, Hsiang-Wen Tseng

**Affiliations:** 1 Center of Excellence for Drug Development, Biomedical Technology and Device Research Labs, Industrial Technology Research Institute, No. 321, Sec 2, Kuangfu Rd, Hsinchu City, 30011 Taiwan; 2 Greenhouse System Technology Center, Industrial Technology Research Institute, Central Region Campus, No.2 Wenxian Rd., Nantou City, 54041 Taiwan; 3 Present address: Bldg 13, No. 321, Sec 2, Kuangfu Rd, Hsinchu City, 30011 Taiwan

**Keywords:** *Juniperus chinensis*, Hepatocellular carcinoma, Antiangiogenesis, Therapeutic agent

## Abstract

**Background:**

To identify a novel therapeutic agent for hepatocellular carcinoma (HCC), for which no promising therapeutic agent exists, we screened a panel of plants and found that *Juniperus chinensis* exhibited potential antiangiogenic and anti-HCC activities. We further investigated the antiangiogenic and anti-HCC effects of the active ingredient of *J. chinensis* extract, CBT-143-S-F6F7, both in vitro and in vivo.

**Methods:**

A tube formation assay conducted using human umbilical vein endothelial cells (HUVECs) was first performed to identify the active ingredient of CBT-143-S-F6F7. A series of angiogenesis studies, including HUVEC migration, Matrigel plug, and chorioallantoic membrane (CAM) assays, were then performed to confirm the effects of CBT-143-S-F6F7 on angiogenesis. The effects of CBT-143-S-F6F7 on tumor growth were investigated using a subcutaneous and orthotopic mouse model of HCC. In vitro studies were performed to investigate the effects of CBT-143-S-F6F7 on the cell cycle and apoptosis in HCC cells. Moreover, protein arrays for angiogenesis and apoptosis were used to discover biomarkers that may be influenced by CBT-143-S-F6F7. Finally, nuclear magnetic resonance analysis was conducted to identify the compounds of CBT-143-S-F6F7.

**Results:**

CBT-143-S-F6F7 showed significantly antiangiogenic activity in various assays, including HUVEC tube formation and migration, CAM, and Matrigel plug assays. In in vivo studies, gavage with CBT-143-S-F6F7 significantly repressed subcutaneous Huh7 tumor growth in severe combined immunodeficient (SCID) mice, and prolonged the survival of orthotopic Huh7 tumor-bearing SCID mice (a 40 % increase in median survival duration compared with the vehicle-treated mice). Immunohistochemical staining of subcutaneous Huh7 tumors in CBT-143-S-F6F7-treated mice showed a significantly decrease in the cell cycle regulatory protein cyclin D1, cellular proliferation marker Ki-67, and endothelial marker CD31. CBT-143-S-F6F7 caused arrest of the G2/M phase and induced Huh7 cell apoptosis, possibly contributing to the inhibition of HCC tumors. Protein array analysis revealed that several angiogenic and antiapoptotic factors were suppressed in CBT-143-S-F6F7-treated Huh7 cells. Finally, five compounds from CBT-143-S-F6F7 were identified.

**Conclusions:**

According to these results, we report for the first time the antiangiogenic and anti-HCC activities of CBT-143-S-F6F7, the active fractional extract of *J. chinensis*. We believe that CBT-143-S-F6F7 warrants further evaluation as a new anti-HCC drug.

**Electronic supplementary material:**

The online version of this article (doi:10.1186/s12906-016-1250-6) contains supplementary material, which is available to authorized users.

## Background

In 2012, hepatocellular carcinoma (HCC) was the sixth most common cancer worldwide. Moreover, in 2012, there were 746,000 liver-cancer-related deaths and 782,000 new HCC cases [1]. Many factors, such as hepatitis B and C virus infection, chronic alcohol consumption, aflatoxin-B1-contaminated food consumption, fatty liver diseases, cirrhosis-inducing conditions, and metabolic diseases, increase the risk of developing HCC [1, 2]. Although many reliable methods could be used in HCC diagnosis, treatments for HCC remain limited. In patients with early-stage HCC, surgical resection and liver transplantation are often used, and the 5-year survival rate can exceed 50 % [3, 4]. However, the recurrence rate in patients who underwent hepatectomy exceeded 50 % in 2 years [5]. Moreover, for patients who are unable to undergo hepatectomy, liver transplantation could be considered, but is effective only for small, unresectable HCCs in patients with cirrhosis [6]. For patients with intermediate-stage HCC, transarterial chemoembolization (TACE) is often performed. TACE results in an improved 2-year survival rate compared with that of patients not suitable for resection, transplant, or radiofrequency ablation [7]. However, only an approximately 40 % complete response rate was reported after TACE treatment [8]. In patients with advanced-stage HCC, systemic chemotherapy could be considered but is not recommended because of the low response rate [1]. Recently, sorafenib, the first drug applied in targeted therapy for HCC, was approved and drew considerable attention. However, sorafenib, on average, could improve the life expectancy of patients by only 2.8 months, which is far from ideal [9]. Thus, developing novel therapeutics for HCC is necessary.

HCC is usually an angiogenic tumor with a strong angiogenesis-inducing ability [10, 11]. Angiogenesis and vascularization can enhance tumor growth and progression by providing a higher nutrient and oxygen supply than that provided through simple nutrient and oxygen diffusion. In addition, angiogenesis and vascularization enable tumor cells to escape into the circulation and easily lodge in other organs, leading to metastatic tumors [12, 13]. New vessels in tumors usually have abnormal structures and functions, leading to an abnormal tumor microenvironment. The abnormal tumor microenvironment, including vascular hyperpermeability, extracellular matrix remodeling, and endothelial cell activation, may cause malignancies that are more aggressive [11]. Because of these findings, new anticancer drug development is targeted toward angiogenesis [14]. Many factors, including vascular endothelial growth factor (VEGF), platelet-derived growth factor (PDGF), basic fibroblast growth factor (FGF), angiogenin, chemokine (C-X-C motif) ligand 16 (CXCL16), PDGF-AA, placental growth factor (PlGF), and urokinase-type plasminogen activator (uPA), contribute to tumor angiogenesis [15–20]. For targeting these molecules, many drugs, such as sorafenib, an oral multitargeted tyrosine kinase inhibitor of VEGF receptor (VEGFR)-1, VEGFR-2, and VEGFR-3 and PDGF receptors, have been developed and benefit HCC patients [21].

*Juniperus chinensis*, commonly known as Chinese juniper, is a native and widely used ornamental plant in East Asian countries. *J. chinensis* and plants of the same genus exhibit many bioactivities, such as antimicrobial, antifungal, antiviral, antiinsect, antifertility, vasorelaxing, and antitumor activities [22]. In in vitro studies, fresh *J. chinensis* leaf extracts exhibited cytotoxicity toward HeLa (human cervical carcinoma) and HGC-27 (human gastric carcinoma) cells [22]. Moreover, topically applying a *J. chinensis* extract inhibited 7,12-dimethylbenz[a]anthracene (DMBA)- and 12-O-tetradecanoylphorbol-13-acetate (TPA)-induced papilloma formation in mice [22]. Widdrol, an odorous compound extracted from *J. chinensis*, inhibited the in vitro growth of human colon adenocarcinoma HT29 cells [23]. The authors demonstrated that widdrol also caused cell cycle arrest in the G1 phase, which is associated with Chk2 induction, p53 phosphorylation, and cyclin-dependent kinase (CDK) inhibitor p21 expression as well as cyclin E, CDK2, and retinoblastoma protein inhibition.

Although *J. chinensis* and its related compounds have exhibited antitumor activities, the effects of *J. chinensis* on the angiogenesis and progression of HCC remain uninvestigated. In this study, the separation and identification of the active ingredient of *J. chinensis* from an extract solution was guided using antiangiogenic and anti-HCC activities. Through a series of in vitro and in vivo preclinical experiments, we first demonstrated that the active ingredient CBT-143-S-F6F7 exhibits antiangiogenic and anti HCC activities. Moreover, we report for the first time that gavage with CBT-143-S-F6F7 prolonged the survival of orthotopic HCC-bearing mice. These results suggest that CBT-143-S-F6F7 is a potential therapeutic agent for HCC.

## Methods

### Plant collection

The raw material of *J. chinensis* was collected in Hsinchu City (GPS coordinate: 24°47'58.4"N 120°59'13.0"E). The raw material was further authenticated as *J. chinensis* L. var. *sargentii* Henry using internal transcribed spacers (ITS) of nuclear ribosomal DNA, and the ITS sequence had been deposited in the GenBank data base (Accession Number: KX496336). A voucher specimen was deposited at the plant collection bank of Industrial Technology Research Institute (ITRI,Hsinchu, Taiwan).

### Preparation of CBT-143-S-F6F7 fraction from *J. chinensis*

Dried thin branches and leaves of *J. chinensis* L. var. *sargentii* Henry were immersed in an 8- to 10-fold weight of 95 % ethanol. Furthermore, for obtaining an extract solution, the immersed plant materials were extracted at the boiling point of the solvent for 1 h. The extract solution was then filtered using filter paper and evaporated to dryness in vacuum. The extract was further separated using a column (inner diameter: 5.5 cm; length: 30 cm), which contained a 15-fold weight of silica gel (0.040–0.063 mm, Silica gel 60, Merck), by using an acetone:n-hexane mixture (from1:4 to 1:1) as the eluent. After separation, the CBT-143-S-F6F7 fraction was identified according to the results of a tube formation assay of human umbilical vein endothelial cells (HUVECs).

### Cell culture

Five HCC cell lines, Huh7, Hep3B, PLC/PRF/5, SK-Hep-1, and HepG2, were used. Huh7 was obtained from the Japanese Collection of Research Bioresources. Hep3B, PLC/PRF/5, and SK-Hep-1 were purchased from the American Type Culture Collection. HepG2 cells and HUVECs were obtained from the Bioresource Collection and Research Center, Taiwan. Hep3B cells were maintained in Eagle’s minimum essential medium supplemented with 10 % heat-inactivated fetal bovine serum (FBS), 100 U/mL penicillin, and 100 μg/mL streptomycin. The other HCC cells were grown in Dulbecco’s minimum essential medium supplemented with 10 % FBS, 100 mM nonessential amino acids, 100 U/mL penicillin, and 100 μg/mL streptomycin. All cells were cultured in a humidified incubator at 37 °C and 5 % CO_2_. All reagents for cell culture were purchased from Life Technologies (Grand Island, NY, USA).

### Animals

Female BALB/c (BALB/cAnNCrlBltw) and SCID (CB17/Icr-Prkdc^scid^/CrlBltw) mice, aged 6–8 weeks and weighing 16–22 g, were purchased from BioLASCO Taiwan Co. Ltd. (Ilan, Taiwan). One week before the study, all mice were housed in conventional cages in the Animal Laboratory for Biomedical Research (fully accredited by the Association for Assessment and Accreditation of Laboratory Animal Care in 2011) of Industrial Technology Research Institute and allowed to acclimatize and recover from shipping-related stress. Sterilized water and food were provided *ad libitum*, and the mice were housed in rooms maintained at 22 °C–26 °C with 40–70 % humidity and a controlled 12-h light–dark cycle. All experimental procedures for animal studies were approved by the Institutional Animal Care and Use Committees of ITRI (subcutaneous xenograft study: ITRI-IACUC-2010-009 V1; Matrigel plug assay: ITRI-IACUC-2010-058 V1; orthotopic model: ITRI-IACUC-2011-020, and chorioallantoic membrane assay [CAM]: ITRI-IACUC-2011-007).

### HUVEC tube formation assay

Tube formation assay was performed as previously described [24]. HUVECs were cultured in growth-factor-reduced Matrigel (BD Biosciences, Franklin Lakes, NJ, USA; 356231) and treated with different CBT-143-S-F6F7 concentrations. The total length of the net structure was calculated using NIS element image analysis software (Nikon; Agent: Lin Trading Co., Ltd. Taiwan). By considering the total length of a net structure formed from a group without drug treatment as 100 %, the state of inhibition of HUVEC net structure formation elicited by different drug treatments was analyzed.

### HUVEC migration assay

The ability of CBT-143-S-F6F7 to inhibit HUVEC migration was observed using a BD transwell system. HUVECs were seeded into an upper chamber in a medium without FBS, and a medium with 10 % FBS was then added to a lower chamber as an attractant. After 24 h, the transwell was collected, and the migrating cells on the lower side of membrane were stained with crystal violet to facilitate observation.

### Matrigel plug assay

Matrigel (BD Biosciences, 354234) was mixed with PBS in a 9:1 ratio for mock control or 500 ng/mL FGF-b (Peprotech, USA, 100-18B) and 500 ng/mL VEGF (R&D, USA, 293-VE-500) for vehicle control. For studying antiangiogenesis, different CBT-143-S-F6F7 concentrations were added to Matrigel mixed with FGF-b and VEGF. Fourteen days after subcutaneous Matrigel injection, BALB/c mice were sacrificed, Matrigel plugs were collected, and hemoglobin in the plugs was quantified using a QuantiChrom Hemoglobin Assay Kit (BioAssay Systems, USA, DIHB-250).

### CAM assay

The embryos of unhatched eggs from specific-pathogen-free white Leghorn chicken were placed laterally in an incubator (37 °C, relative humidity: 55–60 %). On day 4 of the incubation period, 2.5 mL of albumin was drawn from the eggs using a 20-G needle, and a fake air chamber was constructed on the embryo. Subsequently, the needle hole and fake air chamber were sealed using 3 M breathable tape. On day 7, CBT-143-S-F6F7 was dissolved with dimethyl sulfoxide (DMSO) and diluted with PBS. For each group, including a vehicle group, the final DMSO concentration was 1 %. CBT-143-S-F6F7 was loaded on 6-mm-diameter Circular Advantec filter paper (Toyo Roshi Kaisha, Ltd., Tokyo, Japan), and the paper was placed on the CAM. On day 9, the CAM was photographed using the SZX16 dissecting microscope (Olympus, Japan). By using the filter paper as the center, four concentric circles were marked on the photograph (diameters of 7, 8, 9, and 10 mm; total circumference of the four circles was 106.8 mm, and total circumference represented the region near the filter paper) [25]. The amount of vessels crossing the concentric circles (the vascular density index or VDI) was used to evaluate the state of angiogenesis. The VDI for the same photograph was determined by three people, and the mean VDI was adopted.

### Cell viability assay

HCC cells were seeded in 96-well plates at 10^4^ cells/well and treated with CBT-143-S-F6F7 for 48 h. The cells were then exposed to a culture medium containing 0.5 mg/mL of (3-(4,5-dimethylthiazol-2-yl)-2,5-diphenyltetrazolium bromide) (MTT) (Sigma–Aldrich, St. Louis, MO, USA). After 2–3 h, DMSO was added to dissolve the resulting formazan. For each solution, the optical density at 570 nm was measured using a microplate spectrophotometer (SpectraMax M5, Molecular Devices, USA). Cell viability was calculated as follows: viability (%) = A_570_ of treated cells/A_570_ of control cells × 100. The half maximal inhibitory concentration (IC_50_) was estimated using GraFit software.

### In vivo subcutaneous tumor model

Huh7 cells (3 × 10^6^) were suspended in 100 μL of PBS with 25 % Matrigel and subcutaneously implanted into the right flank of female SCID mice. Tumors were measured three times per week by using calipers, and tumor volume was calculated as follows: tumor volume (V) = (LS^2^)/2 (L, longest diameter, mm; S, shortest diameter, mm). Treatments were initiated when the tumor volume reached 150–200 mm^3^. The mice were randomly grouped into groups of six. CBT-143-S-F6F7 was dissolved in a formulation of 10 % 1-Methyl-2-pyrrolidone (M6762, Sigma–Aldrich), 20 % Cremophor EL (C5135, Sigma–Aldrich), and 70 % saline. CBT-143-S-F6F7 was administered daily through gavage at a dose of 100 mg/kg body weight for 21 days. The tumor volume and body weight of the mice were monitored. The antitumor activity of treatments was represented by the percentage of tumor growth inhibition (TGI), which was calculated as follows: [1 − (final tumor volume – initial tumor volume for treated group)/(final tumor volume – initial tumor volume for vehicle group)] × 100. Data are represented as the mean ± standard error of the mean (SEM).

### In vivo orthotopic tumor model

Mice were anesthetized with a mixed solution [40 mg/kg body weight of Zoletil 50 (Virbac, France) and 10 mg/kg body weight of Xylazine (Rompun 2 % injection, Bayer, German)] through intraperitoneal (i.p.) injection. Through an upper middle abdominal cavity incision, the left lateral lobe of the liver was exposed. Huh7 cells (1 × 10^6^) were suspended in 50 μL of PBS with 50 % Matrigel and were implanted into the left lateral lobe of the liver by using an insulin syringe with a needle (29 G X1/2", Terumo, Shibuya, Japan), and the wound was then closed using an interrupted suture and Autoclip wound closing system (59043, Stoelting, USA). Seven days after the implantation, CBT-143-S-F6F7 treatment (100 mg/kg through oral administration) was initiated and proceeded for 42 days. The body weight of the mice was monitored twice a week, and blood samples were collected from the facial vein. Survival analysis was conducted using the Kaplan–Meier method and GraphPad Prism® was used for generating survival curve and performing related statistics. The human α-fetoprotein (AFP) level in mouse serum was determined using AFP ELISA (IB19102, IBL-America, USA).

### Immunohistochemistry analysis

After the mice were sacrificed, tumor tissues were fixed in formalin and embedded in paraffin as tumor sections for further analysis. Immunostaining was completed using an Autostainer Link 48 system (Dako, Glostrup, Denmark). The antihuman Ki-67 (IS-626, Dako), antihuman cyclin D1 (1:500, IS-083, Dako), and anti-CD31 antibodies (LS-B1932, Lifespan, USA) were used for immunostaining. Five fields (100 × 100 μm^2^) of each tumor sample were randomly selected, and the percentages of Ki-67-positive and cyclin D1-positive cells were calculated to evaluate cell proliferation in tumor tissues. For quantifying CD31-positive cells, ten fields of each tumor sample were randomly selected. The results are presented as the mean ± standard deviation (SD).

### Flow cytometry for cell cycle analysis and annexin V staining

For cell cycle analysis, Huh7 cells were seeded in 6-well plates at 2 × 10^5^ cells/well and treated with CBT-143-S-F6F7 for 48 h. The cells were collected through trypsinization and fixed overnight in 70 % ethanol at −20 °C. The fixed cells were then washed with PBS. For measuring DNA content, the cells were stained with a propidium iodide (PI) solution [2 μg/mL PI, 200 μg/mL RNase A, and 0.1 % (w/v) Triton X-100] (Sigma–Aldrich) and incubated in the dark for 30 min at 37 °C. DNA content was determined using a FACScan flow cytometer (BD Biosciences). Data were analyzed using ModFit LT software (Verity Software House, USA).

For apoptosis analysis by using PI and annexin V staining, Huh7 cells were seeded in 6-well plates at 2 × 10^5^ cells/well and treated with CBT-143-S-F6F7 for 48 h. The cells were collected through trypsinization, and PI and annexin V staining was then conducted using an annexin V–fluorescein isothiocyanate apoptosis detection kit (Strong Biotech Corp., Taipei, Taiwan).

### Protein array analysis

Huh7 cells were treated with CBT-143-S-F6F7 for 48 h, and the cell lysate and condition medium were collected and analyzed using the Proteome Profiler Human Apoptosis Array Kit (R&D, ARY009) and Proteome Profiler Human Angiogenesis Array Kit (R&D, ARY007), respectively. The operating procedures were performed according to instructions in the user manual. Chemoluminescent signals were monitored using the BioSpectrum 610 imaging system (UVP, USA) and analyzed using ImageJ software.

### Identification of active compounds from CBT-143-S-F6F7

CBT-143-S-F6F7 was further purified using a fast protein liquid chromatography (FPLC) system, which was conducted using GE Healthcare Unicorn 5.1 controller equipped with P-900 pump, superloop 50 mL, UV-900 monitor detector and Frac-920 fraction collector. FPLC was performed with Toyo Pearl® HW-40S column (inner diameter: 3 cm; length: 30 cm; flow rate: 5 mL/min) and then eluted with 95 % ethanol to yield five fractions. Fraction 2 was further purified using a column (inner diameter: 2 cm; length: 20 cm) packed with 100 g silica gel (0.015-0.040 mm, Silica gel 60, Merck) and then eluted with Ethyl acetate/n-hexane (1:5) at flow rate of 10 mL/min to yield deoxypodophyllotoxin, acetyl podophyllotoxin and yatein. Fraction 3 was further purified using a column (inner diameter: 2 cm; length: 25 cm) packed with 120 g silica gel (0.015-0.040 mm, Silica gel 60, Merck) and eluted with ethyl acetate/n-hexane (1:3) at flow rate of 10 mL/min to get savinin. Fraction 4 was further purified using a column (inner diameter: 2 cm; length: 25 cm) packed with 120 g silica gel (0.015-0.040 mm, Silica gel 60, Merck) and then eluted with ethyl acetate/n-hexane (1:3) at flow rate of 10 mL/min to obtain oxohinokinin. The structures of these compounds were identified using nuclear magnetic resonance (NMR) analysis. ^1^H (600 MHz) and ^13^C (150 MHz) NMR spectra, DEPT, ^1^H-^1^H COSY, NOESY, HSQC, and HMBC experiments were recorded by a varian unity inova 600 NMR spectrometer at room temperature. Chemical shifts were reported in δ units and coupling constants (*J*) in Hz.

To determine the content of oxohinokinin, savinin, deoxypodophyllotoxin, acetyl podophyllotoxin, and yatein in CBT-143-S-F6F7, high performance liquid chromatography (HPLC) was conducted by using a Waters system (Waters 600E controller and Waters 717 plus autosampler) equipped with 996 photodiode array (PDA) detector (Waters, 2998). HPLC was performed by using a Cosmosil 5C18-MS-II C-18 reversed phase column (250 mm x 4.6 mm, 5 μm diameter particles) and the flow rate is 0.8 mL/min. Eluents in reservoirs A-C were as follows: 0.1 % phosphoric acid in water (A), acetonitrile (B), methanol (C). Delivery programs f linear gradient are A/B/C = 65/25/10 to A/B/C = 25/60/15 for 60 min. UV absorption spectra were recorded at 280 nm.

### Statistical analysis

All experimental data are expressed as the mean ± SD, unless otherwise stated. The significance of differences between groups was analyzed using the Student’s *t* test. *P* < 0.05 was considered statistically significant.

## Results

### CBT-143-S-F6F7 is an active ingredient of *J. chinensis* extract inhibiting angiogenesis

**Fig. 1:**
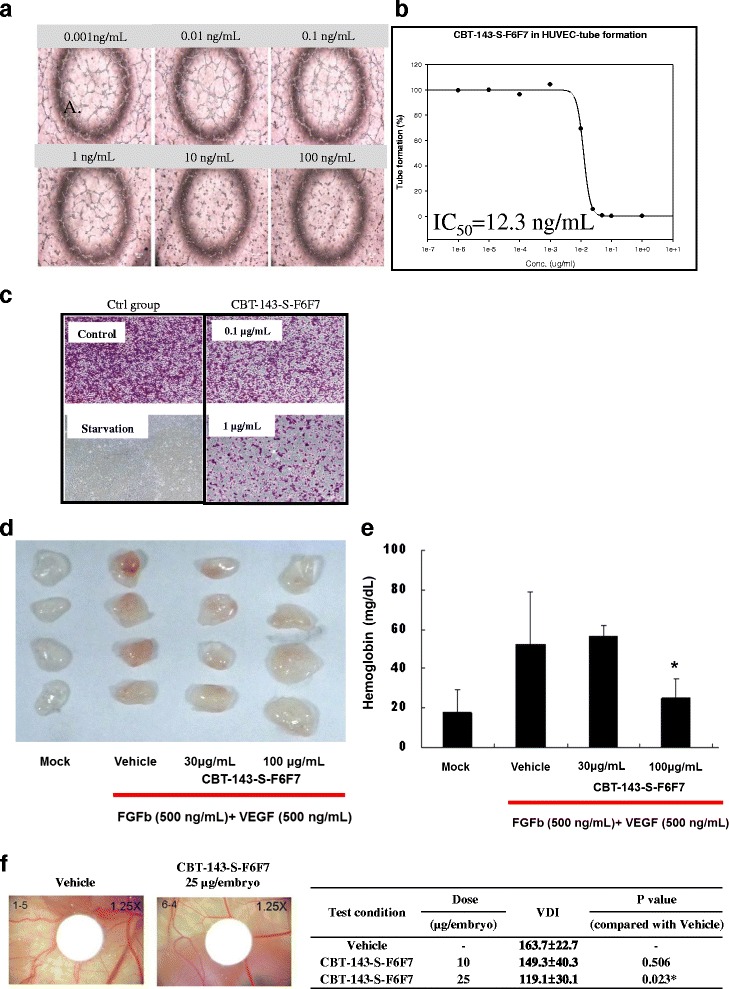
CBT-143-S-F6F7 inhibited angiogenesis. **a** CBT-143-S-F6F7 showed concentration-dependent inhibition the tube formation of HUVECs. **b** The net structure of HUVECs and the related total length of the net structure of HUVECs suppressed by CBT-143-S-F6F7 concentration-dependently. **c** CBT-143-S-F6F7 suppressed HUVEC migration by using a transwell assay. When treated with CBT-143-S-F6F7, less HUVECs stained with crystal violet were observed on lower side of membrane. **d** CBT-143-S-F6F7 inhibited angiogenesis in a Matrigel plug assay (Formation of red color in a Matrigel plug indicate vascular formation.) **e** CBT-143-S-F6F7 concentration-dependently inhibited the content of hemoglobin in the Matrigel plugs.*:*P* < 0.05 vs. vehicle group. **f** CBT-143-S-F6F7 concentration-dependently suppressed vessel formation in a CAM assay. The quantitative analysis of vessel formation was demonstrated using VDI as mentioned in Methods. *:*P* < 0.05 vs. vehicle group

For identifying new drugs that can inhibit angiogenesis, a tube formation assay was performed using HUVECs to screen a panel of extracts from different plants. To quantify the net structure of HUVECs, the total length of the net structure was calculated using NIS element image analysis software. We observed that the crude extract from *J. chinensis* L. var. sargentii Henry, named CBT-143-S, strongly inhibited tube formation in HUVECs, and the IC_50_ was 221 ng/mL (data not shown). To determine the active ingredient of CBT-143-S, different CBT-143-S fractions were separated and collected using a silica gel column. Among these fractions, the fraction CBT-143-S-F6F7 strongly inhibited tube formation in HUVECs (data not shown and Fig. 1a). Moreover, the IC_50_ of CBT-143-S-F6F7 in inhibiting tube formation in HUVECs was 12.3 ng/mL (Fig. 1b), which is 20.3-fold lower than that of CBT-143-S. No cytotoxicity was observed in the CBT-143-S-F6F7-treated group (data not shown). According to the aforementioned data, CBT-143-S-F6F7 was identified as the active ingredient of CBT-143-S and its antiangiogenic efficacy was further investigated using different models. In addition to tube formation inhibition in HUVECs, CBT-143-S-F6F7 also decreased the number of HUVECs on lower side of membrane in a transwell study (Fig. 1c), suggesting CBT-143-S-F6F7 also suppressed HUVEC migration. No cytotoxicity was evident in the CBT-143-S-F6F7-treated group (data not shown). According to these in vitro results, CBT-143-S-F6F7 showed potential inhibitory activities against the tube formation and migration of HUVECs.

To determine whether CBT-143-S-F6F7 can suppress angiogenesis in vivo, a Matrigel plug assay was performed. The Matrigel was mixed with VEGF, and FGFb exhibited a red color, indicating vascularization in Matrigel plugs (Fig. 1d). In the presence of CBT-143-S-F6F7, the Matrigel plugs showed less red color than the vehicle group did, indicating no vascularization in the Matrigel plugs (Fig. 1d). Hemoglobin content in the plugs was measured to quantify the degree of angiogenesis. The hemoglobin content in the Matrigel plugs of the vehicle group was 52.64 ± 26.18 mg/dL. The hemoglobin contents in the Matrigel plugs of 30 μg/mL and 100 μg/mL CBT-143-S-F6F7-treated groups were 56.70 ± 5.58 mg/dL and 25.08 ± 9.59 mg/dL, respectively. This result suggested that CBT-143-S-F6F7 inhibited neovascularization in the Matrigel plug assay (Fig. 1e).

To confirm the in vivo antiangiogenic activity of CBT-143-S-F6F7, a CAM assay was performed. CBT-143-S-F6F7 was loaded onto filter paper at two doses (10 μg and 25 μg per embryo), and the filter paper was placed on the CAM. After treatment for 48 h, the CAM was photographed and the degree of angiogenesis was quantified using the VDI, as described in Subsection of material and methods. The degree of vascularization was lower in the CBT-143-S-F6F7-treated group than in the vehicle-treated group (Fig. 1f). The VDI of the vehicle group was 163.7 ± 22.7 and that of the CBT-143-S-F6F7-treated group was 119.1 ± 30.1 and 149.3 ± 40.3 at doses of 10 μg and 25 μg per embryo, respectively. These results suggested that CBT-143-S-F6F7 reduced the vascularization of the CAM. In summary, CBT-143-S-F6F7 was the active ingredient of the *J. chinensis* extract (CBT-143-S) and inhibited angiogenesis both in vitro and in vivo.

### CBT-143-S-F6F7 inhibits HCC cell growth

According to the aforementioned data, we demonstrated that CBT-143-S-F6F7 was the active ingredient of the *J. chinensis* extract that inhibited angiogenesis, and *J. chinensis* extracts inhibit cancer cell growth [22]. We then investigated whether CBT-143-S-F6F7 inhibited the growth of HCC cells, namely Huh7, Hep3B, HepG2, PLC/PRF/5, and SK-Hep-1 cells. After CBT-143-S-F6F7 treatment for 48 h, the growth-inhibiting effect of CBT-143-S-F6F7 on HCC cells was evaluated using an MTT assay and the IC_50_ of CBT-143-S-F6F7 for different HCC cell types was obtained (Table 1). Among the cell types, Hep3B cells had the lowest sensitivity to CBT-143-S-F6F7. Hep3B cells also have been reported to show lower sensitivity to curcumin and sorafinib when compared with other HCC cells [26, 27]. Checkpoint kinase 1 (Chk1) and growth arrest DNA damage-inducible gene 45 gamma (GADD45γ) have been reported to play roles in this phenomenon respectively [26, 27]. However, because these HCC cell lines inherit different gene heterogeneity, why Hep3B cells had the lowest sensitivity to CBT-143-S-F6F7 may be complicated.

**Table 1:** CBT-143-S-F6F7 inhibited the growth of different HCC cells

IC_50_ (μg/mL)					
Cell line	Huh7	Hep3B	HepG2	PLC/PRF/5	SK-Hep-1
CBT-143-S-F6F7	<0.1	15.71	<0.1	<0.1	<0.1

### CBT-143-S-F6F7 suppresses tumor growth in vivo

**Fig. 2:**
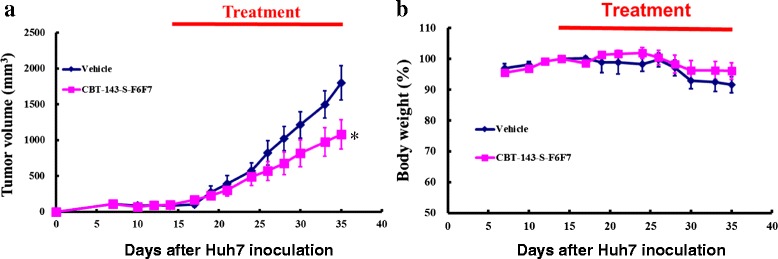
CBT-143-S-F6F7 suppressed the growth of subcutaneous Huh7 tumors in SCID mice. **a** CBT-143-S-F6F7 (100 mg/kg/day) suppressed the growth of Huh7 tumor. *:*P* < 0.05 vs. vehicle group. **b** There was no statistical difference in the body weights of either group of mice throughout the study. In each group, values represent the mean ± SEM. *n* = 7 per group

Because CBT-143-S-F6F7 suppressed angiogenesis and HCC cell growth in vitro, we investigated the in vivo effects of CBT-143-S-F6F7 on Huh7 tumor growth. For 21 days, we administered CBT-143-S-F6F7 through gavage (100 mg/kg/day) to subcutaneous Huh7 tumor-bearing SCID mice. After the treatment, the mean tumor volume of CBT-143-S-F6F7-treated mice was 1081 ± 207 mm^3^ and that of vehicle-treated mice was 1807 ± 240 mm^3^ (Fig. 2a). Tumor growth was significantly repressed in CBT-143-S-F6F7-treated mice compared with vehicle-treated mice (*P* = 0.04). After 21 days of exposure to CBT-143-S-F6F7, the TGI in tumor-harboring mice was 42 % ± 11 %. During CBT-143-S-F6F7 treatment, the mean body weight of CBT-143-S-F6F7-treated mice was not significantly different from that of vehicle-treated mice and no cytotoxicity was observed (Fig. 2b). In summary, these results showed that CBT-143-S-F6F7 suppressed tumor growth without severe adverse effects in Huh7 tumor-bearing mice.

**Fig. 3:**
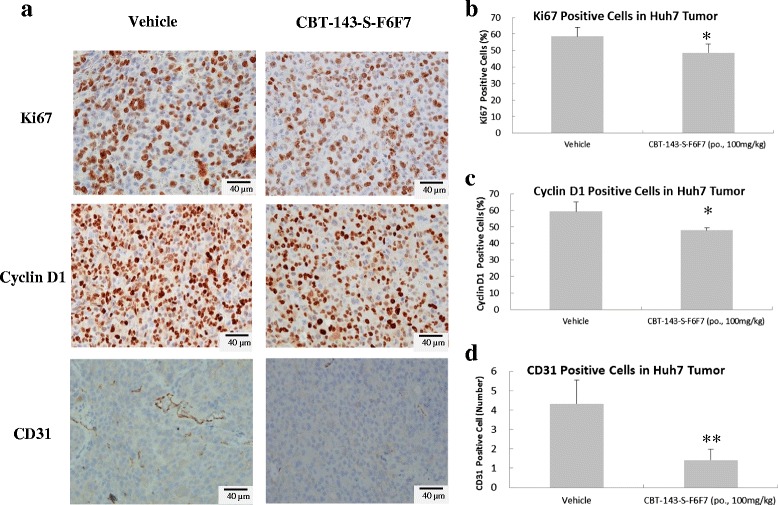
CBT-143-S-F6F7 inhibited Ki67, cyclin D1, and CD31 expression in a Huh7 tumor. **a** Immunohistochemical staining was performed to examine Ki67, cyclin D1, and CD31 expression. **b** The percentage of Ki67 positive cells per field. **c** The percentage of cyclin D1-positive cells per field. **d** The number of CD31-positive cells per field. Data was expressed as the mean ± SD per field. *: *P* < 0.05 compared with vehicle tumors, **: *P* < 0.01 compared with vehicle tumors

The expression of the cell proliferation markers Ki67 and cyclin D1 in Huh7 tumor tissues was analyzed using immunohistochemical analysis (Fig. 3a). In immunohistochemical staining for Ki-67, the percentage of Ki-67-positive cells in CBT-143-S-F6F7-treated tumors was 48.8 ± 5.3 %, which is significantly lower than that in vehicle-treated tumors (58.6 % ± 4.7 %, *P* < 0.05, Fig. 3b). Similarly, the percentage of cyclin D1-positive cells in CBT-143-S-F6F7-treated tumors was 48.0 % ± 1.4 %, which is significantly lower than that in vehicle-treated tumors (59.4 % ± 5.7 %, *P* < 0.05, Fig. 3c). These results collectively showed that CBT-143-S-F6F7 strongly suppressed Ki-67 and cyclin D1 expression in tumors, evidencing that CBT-143-S-F6F7 can inhibit in vivo tumor cell proliferation.

Moreover, to determine whether CBT-143-S-F6F7 can inhibit angiogenesis in tumors, we performed CD31 immunohistochemical staining of Huh7 tumors (Fig. 3a) because CD31 is a vascular endothelial cell marker. The mean number of CD31-positive cells per 100 × 100 μm^2^ in the vehicle group was 4.3 ± 1.2 and that in the CBT-143-S-F6F7-treated group was 1.4 ± 0.5. CBT-143-S-F6F7 significantly reduced the number of vascular endothelial cells (CD31-positive cells) in tumors (*P* < 0.05, Fig. 3d). In summary, CBT-143-S-F6F7 suppressed Huh7 tumor cell proliferation and reduced the number of vascular endothelial cells in Huh7 tumors.

### CBT-143-S-F6F7 prolongs the survival of orthotopic HCC-tumor-bearing mice

**Fig. 4:**
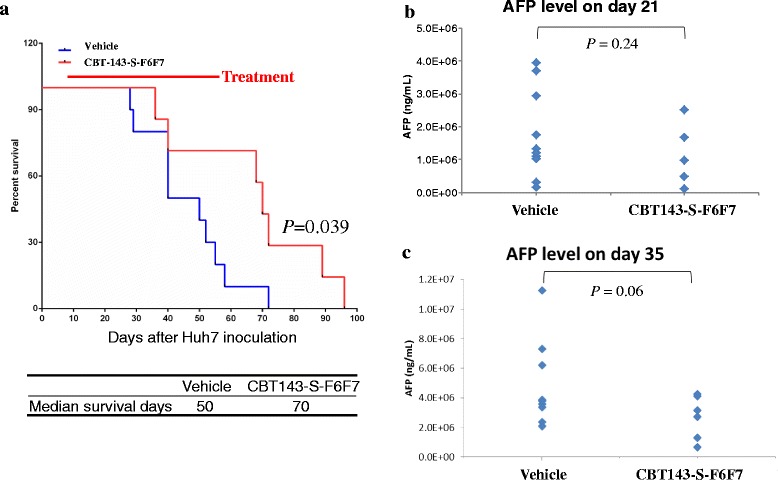
CBT-143-S-F6F7 prolonged the survival of orthotopic Huh7-tumor-bearing mice. **a** Survival curves of orthotopic Huh7- tumor-bearing mice and median survival duration. **b** Serum AFP concentration at days 21 after implanting Huh7 cells. **c** Serum AFP concentration at days 35 after implanting Huh7 cells. Vehicle group, *n* = 10; CBT-143-S-F6F7-treated group, *n* = 7

According to the aforementioned data, CBT-143-S-F6F7 strongly suppressed subcutaneous HCC tumor growth in a mouse xenograft model; however, a subcutaneous HCC tumor is considerably different in the real physiological environment of HCC in the liver. To further evaluate the antitumor activity of CBT-143-S-F6F7, we used an orthotopic HCC model in SCID mice. Seven days after the orthotopic implantation of Huh7 cells in the liver, CBT-143-S-F6F7 was administered daily through gavage until the experiment ended. The survival curves of vehicle- and CBT-143-S-F6F7-treated mice with orthotopic Huh7 tumors are shown in Fig. 4a. The median survival duration of the CBT-143-S-F6F7-treated group was 70 days, which is significantly longer than that of the vehicle-treated group (50 days). This result showed that CBT-143-S-F6F7 prolonged the survival of orthotopic HCC-tumor-bearing mice.

Because AFP is a surrogate biomarker in HCC diagnostics, we performed AFP ELISA to monitor the concentration of AFP, used as an indicator for monitoring orthotopic HCC tumor growth, in orthotopic HCC-tumor-bearing mice. On day 21 after tumor implantation, the AFP concentration in the vehicle group was 1,752,948 ± 1,338,693 ng/mL and that in the CBT-143-S-F6F7-treated group was 1,312,510 ± 944,306 ng/mL (*P* = 0.24, Fig. 4b). Moreover, on day 35 after tumor implantation, the AFP concentration in the vehicle-treated group was 4,780,122 ± 2,929,242 ng/mL and that in the CBT-143-S-F6F7-treated group was 2,705,733 ± 1,463,381 ng/mL (*P* = 0.06, Fig. 4c). At both instances, the AFP concentration in the CBT-143-S-F6F7-treated group was lower than that in the vehicle-treated group; however, the reduction in the AFP concentration was not statistically significant. Nevertheless, the *P* value decreased from 0.24 to 0.07 from days 21 to 35, indicating that the CBT-143-S-F6F7-induced reduction in AFP concentration was more statistically significant in this period. In summary, CBT-143-S-F6F7 suppressed orthotopic Huh7 tumor growth and prolonged the survival of orthotopic Huh7 tumor-bearing mice.

### CBT-143-S-F6F7 causes G2/M arrest and induces apoptosis in HCC cells

**Fig. 5:**
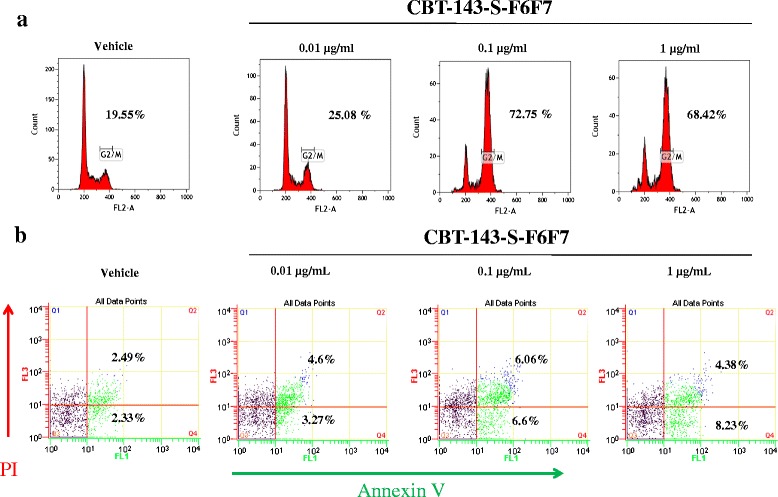
CBT-143-S-F6F7 caused G2/M arrest and induced Huh7 cell apoptosis. **a** CBT-143-S-F6F7 caused G2/M arrest in Huh7 cells in analysis of cell cycle by PI staining for DNA content. **b** The apoptotic status of Huh7 cells was monitored using PI and annexin V staining

To identify factors causing the growth inhibitory effect of CBT-143-S-F6F7 on liver cancer cells, we investigated the effect of CBT-143-S-F6F7 on the HCC cell cycle. Huh7 cells were treated with various CBT-143-S-F6F7 concentrations for 48 h, and the cell cycle state was determined using PI staining and flow cytometry. Obvious G2/M arrest was observed in CBT-143-S-F6F7-treated Huh7 cells (Fig. 5a). The number of G2/M-arrested cells increased after the CBT-143-S-F6F7 concentration increased. These data suggest that CBT-143-S-F6F7-induced inhibition of cell cycle progression could be a molecular event associated with the growth inhibitory effect of CBT-143-S-F6F7 in HCC cells.

To determine whether CBT-143-S-F6F7 can induce Huh7 cell apoptosis, PI and annexin V staining were performed for monitoring the apoptotic status of CBT-143-S-F6F7-treated Huh7 cells. More early apoptotic cells were detected among CBT-143-S-F6F7-treated Huh7 cells, and the percentages of early apoptotic cells were 3.27 %, 6.6 %, and 8.23 % for 0.01, 0.1, and 1 μg/mL CBT-143-S-F6F7, respectively (Fig. 5b). These results suggested that CTB-143-S-F6F7 arrested the cell cycle in the G2/M phase and induced apoptosis in Huh7 cells, possibly contributing to the growth inhibitory effect of CBT-143-S-F6F7 in HCC cells.

### Protein array analysis of CBT-143-S-F6F7-treated Huh7 cells

**Fig. 6:**
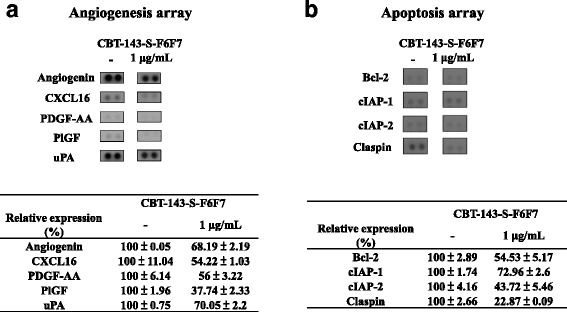
Angiogenesis and apoptosis array analysis of CBT-143-S-F6F7-treated Huh7 cells. **a** Five angiogenesis-related factors were down-regulated when treated with CBT-143-S-F6F7. **b** four apoptotic factors were down-regulated when treated with CBT-143-S-F6F7. The density of each spot was quantified using ImageJ, and the expression of each protein was normalized with that of a vehicle-treated sample

The effects of CBT-143-S-F6F7 on the expression of angiogenic and apoptotic factors in Huh7 cells were studied at the protein level by using the Proteome Profiler Human Angiogenesis Array and Proteome Profiler Human Apoptosis Array (Additional file 1: Figure S1). Figure 6a shows changes in the angiogenesis-related protein expression profile and corresponding protein expression quantified using densitometric analysis after the incubation of Huh7 cells with CBT-143-SF6F7 for 48 h. Five angiogenic factors, angiogenin, CXCL16, PDGF-AA, PlGF, and uPA, were obviously suppressed after CBT-143-SF6F7 treatment, and the results regarding relative expression are shown in Fig. 6a. Changes in the apoptosis-related protein expression profile are shown in Fig. 6b, and four antiapoptotic factors, namely B-cell lymphoma 2 (Bcl-2), cellular inhibitor of apoptosis 1 (cIAP-1), cellular inhibitor of apoptosis 2 (cIAP-2), and Claspin, were suppressed by CBT-143-S-F6F7 treatment. The quantified results are shown as the relative expression in Fig. 6b. Further confirmation of these results and the detailed mechanisms is in progress.

### Identification of compounds from CBT-143-S-F6F7

**Fig. 7:**
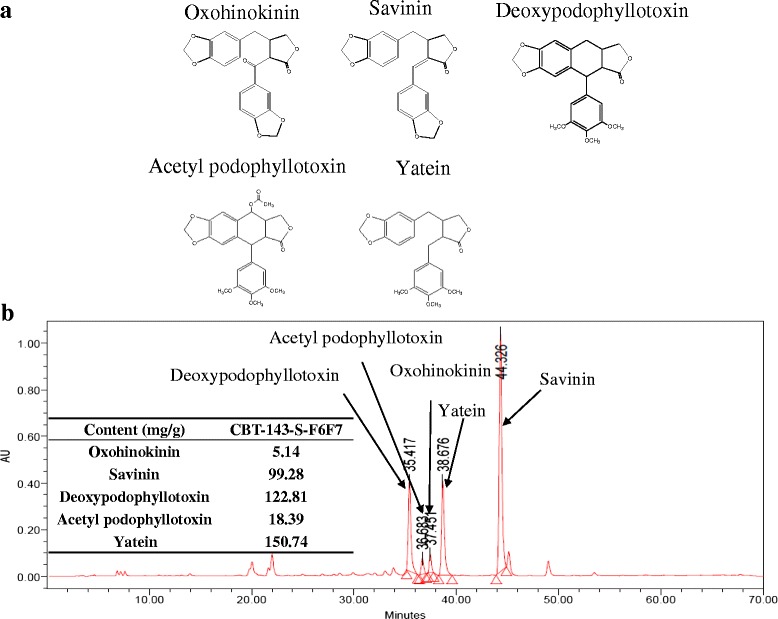
Identification of compounds from CBT-143-S-F6F7. **a** Compounds obtained from CBT-143-S-F6F7. **b** The content of each identified compound in CBT-143-S-F6F7 was determined using high-performance liquid chromatography

According to the aforementioned results, CBT-143-S-F6F7 considerably suppressed angiogenesis and HCC cell growth both in vitro and in vivo. We identified the possible active compounds in CBT-143-S-F6F7. After a series of purification and isolation steps, we identified five compounds from CBT-143-S-F6F7, and their structures were identified by using nuclear NMR analysis and are shown in Fig. 7a. According to the literature, these five compounds are oxohinokinin, savinin, deoxypodophyllotoxin, acetyl podophyllotoxin, and yatein. The contents of these compounds from CBT-143-S-F6F7 were determined using high-performance liquid chromatography (Fig. 7b). The contents of deoxypodophyllotoxin (122.81 mg/g) and yatein (150.74 mg/g) were the highest in CBT-143-S-F6F7. For determining the active compounds of CBT-143-S-F6F7, mass purification and functional studies of these compounds are in progress.

## Discussion

HCC is the sixth most common cancer worldwide in 2012 [1]. HCC patients usually have a poor prognosis because of the advanced stage of HCC at the time of initial diagnosis, unsuitability of patients for surgery, and frequent recurrence and resistance to conventional therapy [3, 4]. HCC is usually an angiogenic cancer with a strong angiogenesis-inducing ability, which could cause more aggressive malignancies [10, 11]. Accordingly, new anticancer drug development is targeted toward angiogenesis [14]. Although many drugs for targeted therapy for HCC have been developed, no promising results have been reported. For example, sorafenib, the first targeted therapy drug for HCC, shows antiangiogenic activities. However, sorafenib only slightly improved life expectancy [9]. To provide an alternative HCC treatment, we used herbal extracts to identify new therapeutic agents targeting angiogenesis. After screening a panel of herb extracts by using a tube formation assay, we identified the major active ingredient of *J. chinensis* extract, CBT-143-S-F6F7, which showed antiangiogenic activity in in vitro and in vivo models, including the inhibition of tube formation and migration of HUVECs and suppression of vascularization in CAM and Matrigel plug assays (Fig. 1). CBT-143-S-F6F7 also repressed the growth of subcutaneous Huh7 tumors in SCID mice (Fig. 2a) and reduced the expression of the cell cycle regulatory protein cyclin D1, cellular proliferation marker Ki-67, and endothelial marker CD31, according to immunohistochemical staining of subcutaneous HCC tumors (Fig. 3). We also demonstrated for the first time that CBT-143-S-F6F7 prolonged the survival of orthotopic HCC-tumor-bearing SCID mice (Fig. 4). Regarding safety, compared with the mice in the vehicle group, mice continually administered CBT-143-S-F6F7 for 21 days did not show a loss of body weight (Fig. 2b) or other side effects, suggesting that CBT-143-S-F6F7 has no significant toxicity in mice. CBT-143-S-F6F7 inhibited the growth of several different HCC cells (Table 1); this inhibition was associated with cell cycle arrest in the G2/M phase and apoptosis induction in Huh7 cells (Fig. 5). Taken together, these results demonstrate that CBT-143-S-F6F7 showed antiangiogenic and anti-HCC activities and could be a potential candidate for HCC therapy.

*J. chinensis* L. extracts can inhibit the growth of some cancer cell types, and topically applying the extracts inhibited DMBA- and TPA-induced papilloma formation in mice [22]. Here, we first report the antiangiogenic and anti-HCC activities of CBT-143-S-F6F7, an active ingredient of a *J. chinensis* L. extract, in in vitro and in vivo studies. Most importantly, we demonstrated that orally administering CBT-143-S-F6F7 suppressed the growth of subcutaneous HCC tumors and prolonged the survival of orthotopic HCC-tumor-bearing SCID mice. The anti-HCC activity of CBT-143-S-F6F7 could be attributed to its effects of angiogenesis inhibition, HCC cell growth suppression, and apoptosis induction in HCC cells. The antiangiogenic effect of CBT-143-S-F6F7 was demonstrated in IHC analysis of a subcutaneous HCC tumor, and CBT-143-S-F6F7 reduced the number of vascular endothelial cells (CD31+ cells), leading to decreased angiogenesis and nutrient supply and tumor growth inhibition. Moreover, CBT-143-S-F6F7 inhibited HCC cell growth (Table 1) by arresting the cell cycle in the G2/M phase (Fig. 5a) and inducing apoptosis (Fig. 5b). However, the effect of CBT-143-S-F6F7-induced apoptosis was weaker than that of CBT-143-S-F6F7-induced cell cycle arrest in the G2/M phase (Fig. 5), possibly suggesting that cell cycle arrest in the G2/M phase may be the main mechanism of CBT-143-S-F6F7 in inhibiting HCC cell growth. Tumor growth inhibition was demonstrated in the IHC analysis of a subcutaneous HCC tumor, and CBT-143-S-F6F7 reduced the expression of the cell proliferation markers Ki67 and cyclin D1 [28, 29]. Collectively, by inhibiting the angiogenic growth of HCC cells, CBT-143-S-F6F7 showed strong in vivo antiHCC effects. Moreover, many anticancer drugs, such as paclitaxel, cause G2/M arrest and induce apoptosis, and many of them are tubulin-targeting drugs [30]. In the present study, CBT-143-S-F6F7 showed similar effects, suggesting that tubulin may be a CBT-143-S-F6F7 target; however, additional studies are required for verifying this assumption.

CBT-143-S-F6F7 has many advantages as a new therapeutic agent for HCC. For example, CTB-143-S-F6F7 could be orally administrated to perform various anticancer activities. Presently, many anticancer drugs, including small molecules and protein drugs, are administrated through infusion, which can be performed only by well-trained medical personnel. Treatment through the oral route is clinically convenient and suitable for cancer patients who usually require prolonged treatment regimens [31]. CBT-143-S-F6F7 is advantageous for clinical use in the future. Moreover, compared with normal cells, cancer cells accumulate many distinct genetic and epigenetic alterations, which affect multiple regulatory pathways within the cells and cause distinct malignant phenotypes. For treating cancer, multitarget drugs are believed to yield more positive outcomes than single-target drugs do. Herbal medicines have drawn more attention recently because of their mixed components, which may have various bioactivities [32]. According to the aforementioned results, CBT-143-S-F6F7 exhibits both antiangiogenic and anticancer activities, reflecting the trend of developing multitarget drugs, and could prove advantageous in treating cancers in the future.

To determine the possible mechanism through which CBT-143-S-F6F7 inhibits angiogenesis and induces apoptosis, the Proteome Profiler Human Angiogenesis Array and Proteome Profiler Human Apoptosis Array were used for identifying CBT-143-S-F6F7-targeted angiogenic and apoptotic factors. Compared with vehicle treatment, we found that five angiogenic factors, angiogenin, CXCL16, PDGF-AA, PlGF, and uPA, were suppressed by CBT-143-S-F6F7 treatment in Huh7 cells. Angiogenin is usually upregulated in various cancers such as colorectal carcinoma, breast cancer, and HCC [33], and it promotes angiogenesis and induces cancer proliferation [16]. CXCL16 and its receptor CXCR6 are closely associated with endothelial progenitor cell recruitment and angiogenesis [17]. PDGF-AA is an angiogenic factor, responsible for the failure of reinitiated interferon (IFN)-α treatment when HCC recurs after the initial IFN-α treatment [18]. PlGF, a VEGF family member, is an attractive drug candidate for angiogenesis inhibition in HCC [19]. uPA is a well-known angiogenic factor in urokinase-mediated plasminogen activation system for modifying the extracellular matrix and is highly involved in angiogenesis [20]. CBT-143-S-F6F7 may inhibit angiogenesis by suppressing these angiogenic factors. Apoptosis array analysis showed that Bcl-2, cIAP-1, cIAP-2, and Claspin were suppressed in CBT-143-S-F6F7-treated Huh7 cells. Bcl-2 protein, an antiapoptotic factor, is often deregulated in cancer and has become a promising anticancer drug target [34]. cIAP-1 and cIAP-2 are inhibitor of apoptosis protein members, which contribute to apoptosis resistance and are typically overexpressed in HCC tissues [35]. Under apoptotic conditions, Claspin is cleaved by caspases 3 and 7 and has antiapoptotic properties [36]. CBT-143-S-F6F7-induced HCC cell apoptosis may be attributed to the inhibition of these antiapoptotic factors. Although a protein array provides a high-throughput method for identifying the possible protein targets of CBT-143-S-F6F7, additional studies are required and are in progress for clarifying the detailed mechanisms.

To determine the possible active compounds of CBT-143-S-F6F7, we performed a series of purification and isolation steps and identified five compounds, oxohinokinin, savinin, deoxypodophyllotoxin, acetyl podophyllotoxin, and yatein. Oxohinokinin was isolated from *Stellera chamaejasme* L. [37], but no further bioactivity has been reported. Savinin induced apoptosis in HCT116 colon carcinoma cells [38]. Deoxypodophyllotoxin has antiproliferative, antitumor, antiinflammatory, antioxidant, antiallergic, and antiangiogenic activities [39, 40]. Moreover, deoxypodophyllotoxin induced apoptosis in breast cancer MDA-MB-231 cells and triggered necroptosis in human non-small-cell lung cancer NCI-H460 cells [41, 42]. Acetyl podophyllotoxin is cytotoxic to various cancer cell lines, such as KB (nasopharyngeal), HF-6 (colon), MCF7 (breast), and PC-3 (prostate) cell lines [43]. Yatein has anti-proliferative activity [44] and is cytotoxic to various cancer cell types, such as DLD-1 (human colorectal carcinoma), CCRF-CEM (human lymphoblastic leukemia), HL-60 (human myeloid leukemia), and IMR-32 cells (human neuroblastoma) [45]. Although some of these compounds show antitumor activity, evidence of their antiangiogenic and anti-HCC activities remains elusive. According to our study results, we first report the anti-HCC and antiangiogenic activities of CBT-143-S-F6F7 and suggest the anti-HCC and antiangiogenic activities of these compounds. An additional study for identifying the active compounds of CBT-143-S-F6F7 is in progress.

## Conclusions

CBT-143-S-F6F7, an active ingredient from an extract of *J. chinensis* L. var. sargentii Henry, showed antiangiogenic activity against the tube formation of HUVECs and in in vivo Matrigel plug and CAM assays. Furthermore, CBT-143-S-F6F7 showed anti-proliferative activity against different HCC cells. It also repressed the growth of Huh7 tumors in SCID mice and prolonged the survival of orthotopic Huh7-tumor-bearing mice. Because of its antiangiogenic and anti-HCC activities, CBT-143-S-F6F7 can potentially be developed as a novel anti-HCC drug.

## Abbreviations

Bcl-2, B-cell lymphoma 2; CAM, chorioallantoic membrane; CDK, cyclin-dependent kinase; cIAP, cellular inhibitor of apoptosis; CXCL16, chemokine (C-X-C motif) ligand 16; FGF, basic fibroblast growth factor; GPS, global positioning system; HCC, hepatocellular carcinoma; IHC, immunohistochemical; PDGF, platelet-derived growth factor; PlGF, placental growth factor; SCID, severe combined immunodeficient; TACE, transarterial chemoembolization; uPA, urokinase-type plasminogen activator; VEGF, vascular endothelial growth factor

## Additional file

Additional file 1: Figure S1. Angiogenesis and apoptosis array analysis of CBT-143-S-F6F7-treated Huh7 cells. (PPT 1587 kb)
