# Supplementary material for: Antiangiogenic and antihepatocellular carcinoma activities of the Juniperus chinensis extract
Source: BMC Complement Altern Med. 2016 Aug 8;16:277. doi: 10.1186/s12906-016-1250-6 (PMC4977662; doi:10.1186/s12906-016-1250-6)
Supplement: Additional file 1: Figure S1. — Angiogenesis and apoptosis array analysis of CBT-143-S-F6F7-treated Huh7 cells. (PPT 1587 kb) [file 12906_2016_1250_MOESM1_ESM.ppt]

## Slide 1
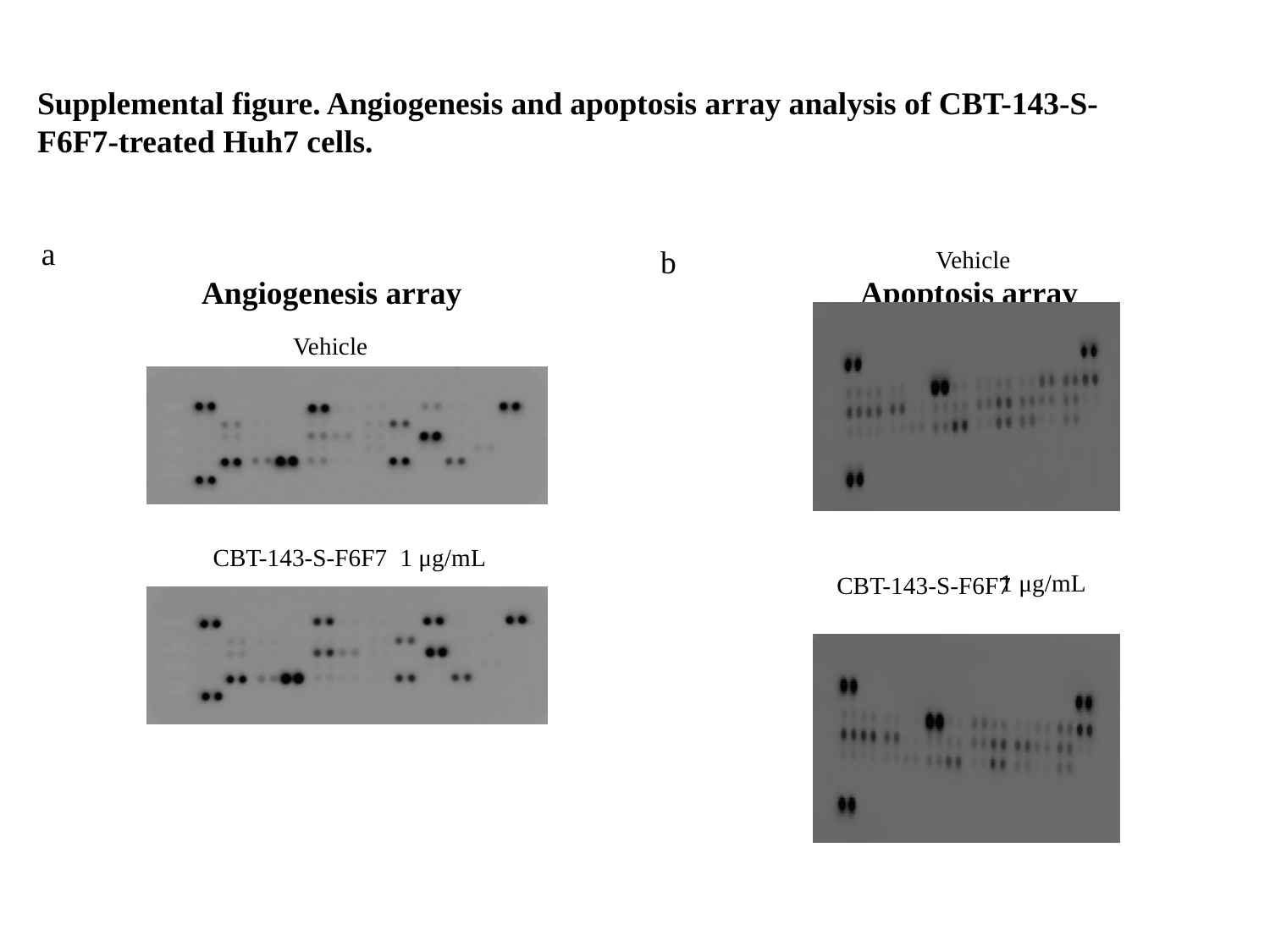

Supplemental figure. Angiogenesis and apoptosis array analysis of CBT-143-S-F6F7-treated Huh7 cells.
a
b
Vehicle
1 μg/mL
CBT-143-S-F6F7
Angiogenesis array
Apoptosis array
Vehicle
CBT-143-S-F6F7
1 μg/mL
